# Supplementary figures and images for: 7C: Computational Chromosome Conformation Capture by Correlation of ChIP-seq at CTCF motifs
Source: BMC Genomics. 2019 Oct 25;20:777. doi: 10.1186/s12864-019-6088-0 (PMC6814980; doi:10.1186/s12864-019-6088-0)

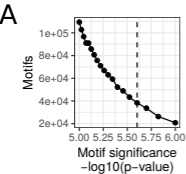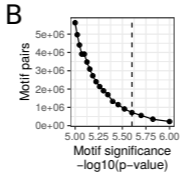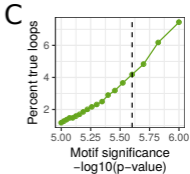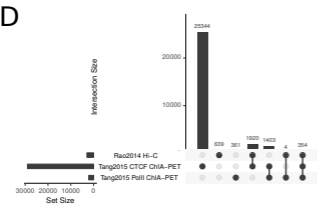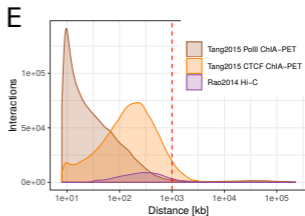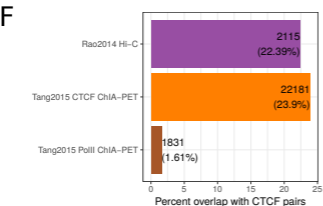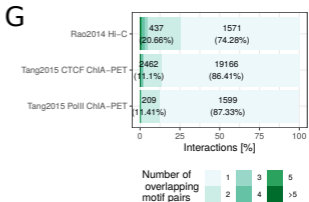

Supplement: Supplementary file 4 — Additional file 4: Figure S1. Hi-C and ChIA-PET interactions and their overlap with CTCF motif pairs. (A) Number of genome-wide CTCF motifs by motif hit significance cutoff. (B) Number of CTCF motif pairs within 1 Mb distance by motif hit significance. (C) Percent of CTCF motif pairs that overlap with experimentally measured Hi-C and ChIA-PET loops by the motif hit significance. (D) Upset plot of true loop data sets (rows) and their size (horizontal bars) with their intersections (columns, and vertical bars) based on the number of overlapping CTCF motif pairs. (E) Distribution of interaction span (distance between anchors) of Hi-C loops and ChIA-PET loops in GM12878 that are used as gold standard. The dotted red line indicates the distance cutoff (1 Mb) used in this study. (F) Number and percent of Hi-C and ChIA-PET loops that overlap with CTCF motif pairs within a distance of 1 Mb. (G) Number and percent of Hi-C and ChIA-PET loops that overlap with 1, 2, 3, 4, 5 or more than 5 CTCF motif pairs. The percent values are relative to all loops that overlap at least one CTCF motif pair. [file 12864_2019_6088_MOESM4_ESM.pdf]

A

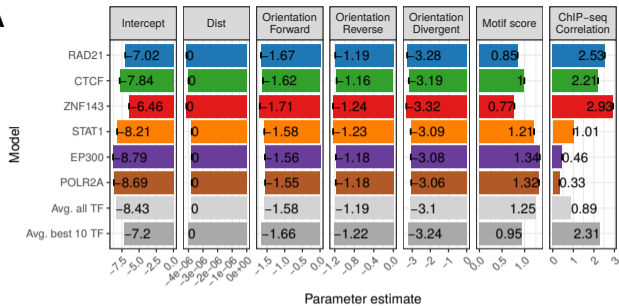

B

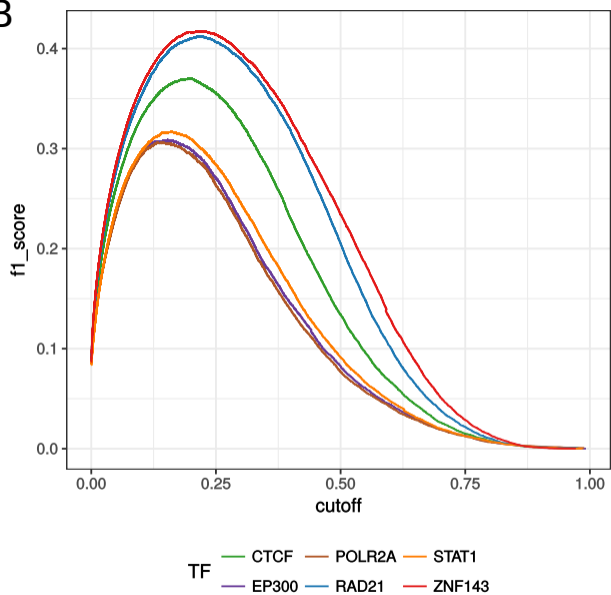

Supplement: Supplementary file 5 — Additional file 5: Figure S2. 7C model parameters and optimal cut-offs for binary prediction. (A) Parameter values of the logistic regression model in 7C for different features (columns), separated for different models (rows). Average of model parameters of model training in 10-fold cross-validation is shown with error bars indicating the standard deviations. While the first six rows represent the models with the indicated TF ChIP-seq data and the genomic features, “Avg. all TF” is the average across all 124 TFs analyzed and “Avg. best 10 TF” is the average across the best ten performing TF models. (B) Prediction performance as f1 score (y-axis) for different cutoffs on the prediction probability p for the six selected models. [file 12864_2019_6088_MOESM5_ESM.pdf]

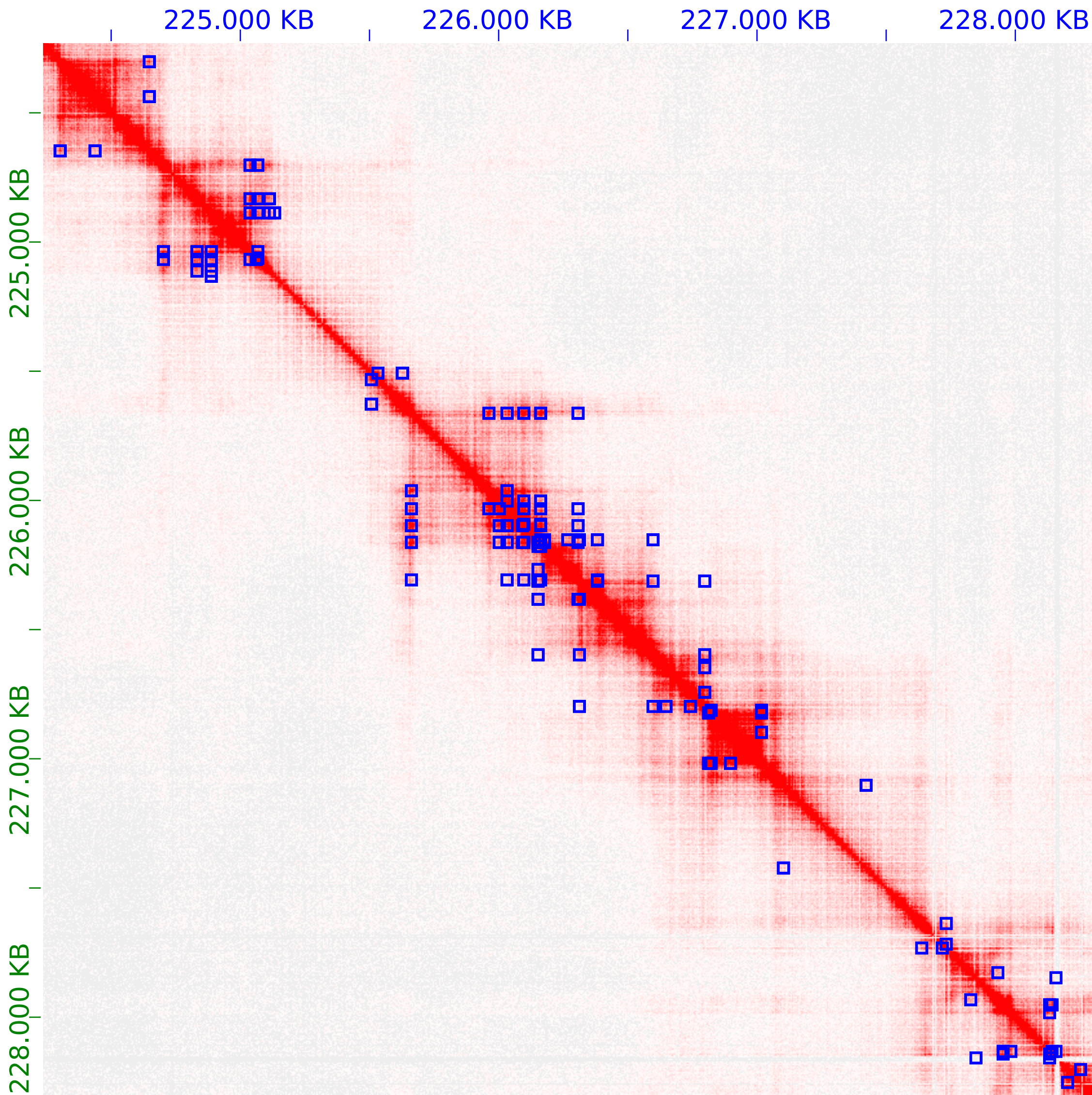

Supplement: Supplementary file 6 — Additional file 6: Figure S3. High resolution Hi-C map with 7C loop predictions. The red color intensity shows Hi-C interaction frequencies at an example locus of chromosome 1. The blue squares indicate 7C loop predictions using a Rad21 ChIP-seq experiment. The figure was created using the Juicebox tool by loading the combined Hi-C data set in GM12878 from [13] with mapping quality MAPQ ≥30 at a resolution of 5 kb. [file 12864_2019_6088_MOESM6_ESM.pdf]

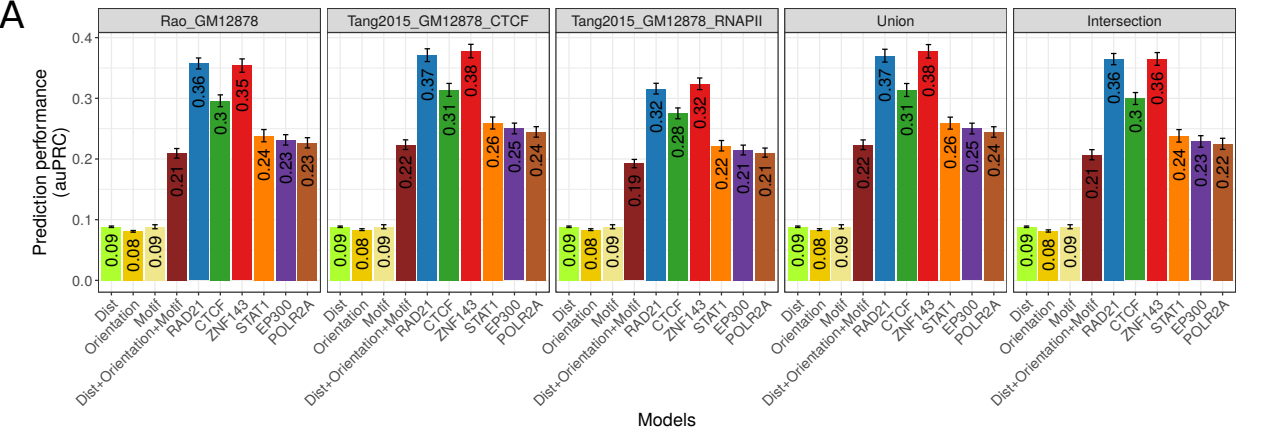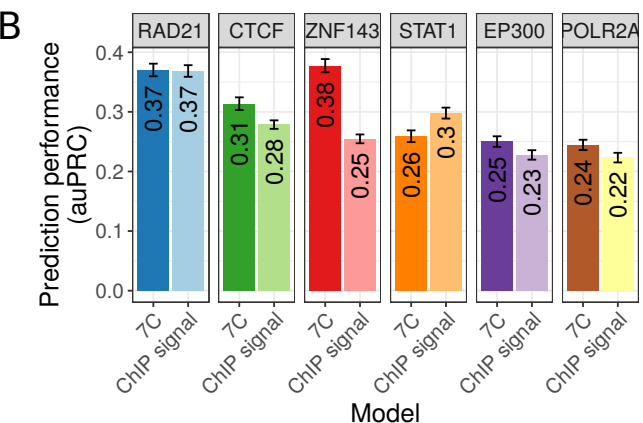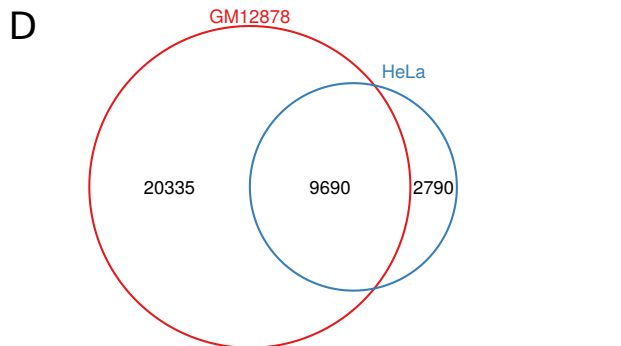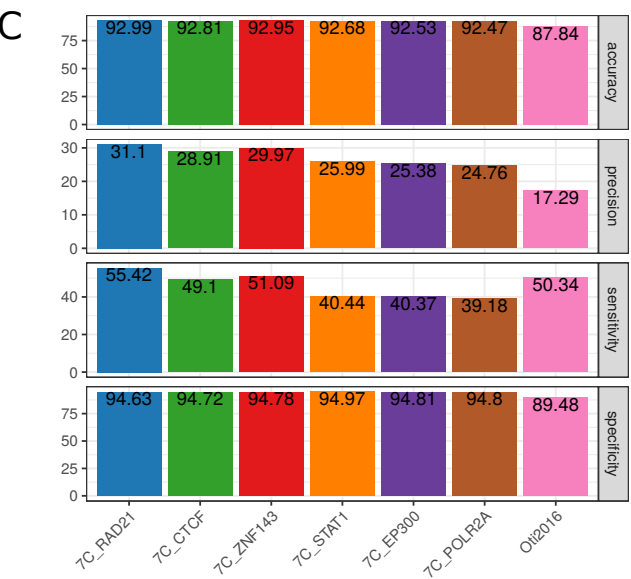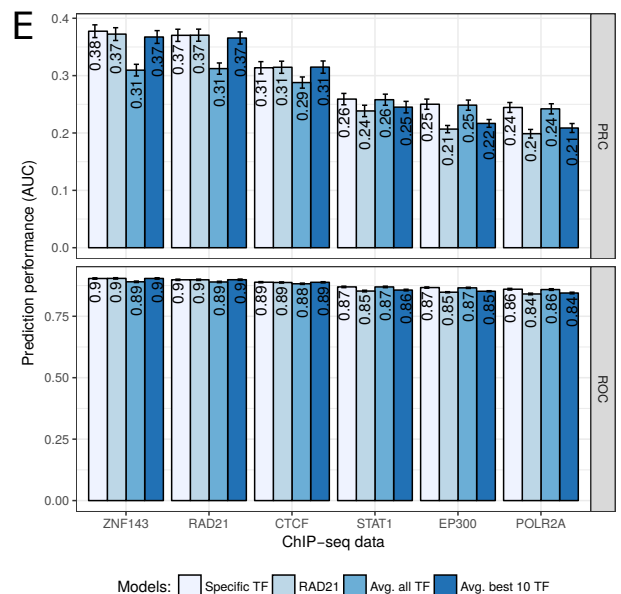

Supplement: Supplementary file 7 — Additional file 7: Figure S4. (A) Prediction performance (auPRC) of 7C when trained and evaluated on different datasets of experimentally measured loops as gold standard. Rao_GM12878 refers to Hi-C loops from [13], Tang2015_GM12878_CTCF, and Tang2015_GM12878_RNAPII to ChIA-PET loops using CTCF or Polymerase II as the target [16]. In Union, all datasets were taken together, and in Intersection, only those CTCF motif pairs that were measured in all datasets were considered positive. (B) Prediction performance (auPRC) of 7C compared to a logistic regression model that uses only the the total coverage signal within +/− 500 bp around the motif center at both loop anchor sites separately. In both models the genomic signal are also included as input features. The performance is shown for six selected TFs in cross-validation on the GM12878 data set. (C) Prediction performance of 7C with six different TFs compared to the method by Oti et al. [40]. The figure shows from top to bottom the accuracy, precision, sensitivity, and specificity of the predictions. (D) Venn-Diagram of CTCF motif pairs overlapping experimentally determined chromatin interactions in GM12878 cells and HeLa cells by Hi-C and ChIA-PET. (E) Prediction performance as auPRC (top) and auROC (bottom) of four different models (colors) on ChIP-seq data for six selected TFs (x-axis). ‘Specific TF’ is the model fitted using the ChIP-seq data indicated on the x-axis, ‘RAD21’ is the model trained on RAD21 ChIP-seq data, ‘Avg. all TF’ is a model averaged across all 124 models of analyzed TFs, and ‘Avg. best 10 TF’ is the averaged model across the 10 best performing models. [file 12864_2019_6088_MOESM7_ESM.pdf]
